# 0027. Kinetic of muscle mass regulation during experimental sepsis

**DOI:** 10.1186/2197-425X-2-S1-O3

**Published:** 2014-09-26

**Authors:** J-C Palao, J Morel, A-C Durieux, J Castells, S Molliex, D Freyssenet

**Affiliations:** Centre Hospitalier Universitaire de Saint-Etienne, Service d'Anesthésie-Réanimation, Saint-Priest en Jarez, France; Université de Lyon, Laboratoire de Physiologie de l'Exercice, Saint-Etienne, France

## Introduction

Sepsis induced muscle weakness (SIMW) is associated with an important morbidity of critical care patients. Moreover, respiratory muscles weakness delays weaning from mechanical ventilation.

The down-regulation of Akt pathway and the activation of proteolytic pathways, such as ubiquitin-proteasome or autophagy-lysosome pathways, could play a key role in the pathogenesis of SIMW. However, the kinetic response of these pathways is currently unknown.

## Objectives

The aim of this study was to describe the kinetic response of anabolic and proteolytic pathways in a murine model of sepsis. A clear picture of mechanisms involved in SIMW could help to develop strategies to prevent muscle casting.

## Methods

Sixteen week-old male mice were divided in a septic group (peritonitis induced by cecal ligature and puncture, CLP) and a sham group (laparotomy without cecal ligature or puncture). Sham-operated mice were pair-fed to septic mice. Following surgery, mice were daily hydrated subcutaneously.

Mice were weighed daily and muscle strength assessed by grip test. Animals were sacrificed at day 1, 4 or 7 after surgery (n = 8/group). Tibialis anterior, gastrocnemius, quadriceps, soleus, extensor digitorum longus and diaphragm muscles were removed to perform molecular (enzymology, RT-qPCR and western blotting) and histological analyses.

## Results

**Figure 1:**
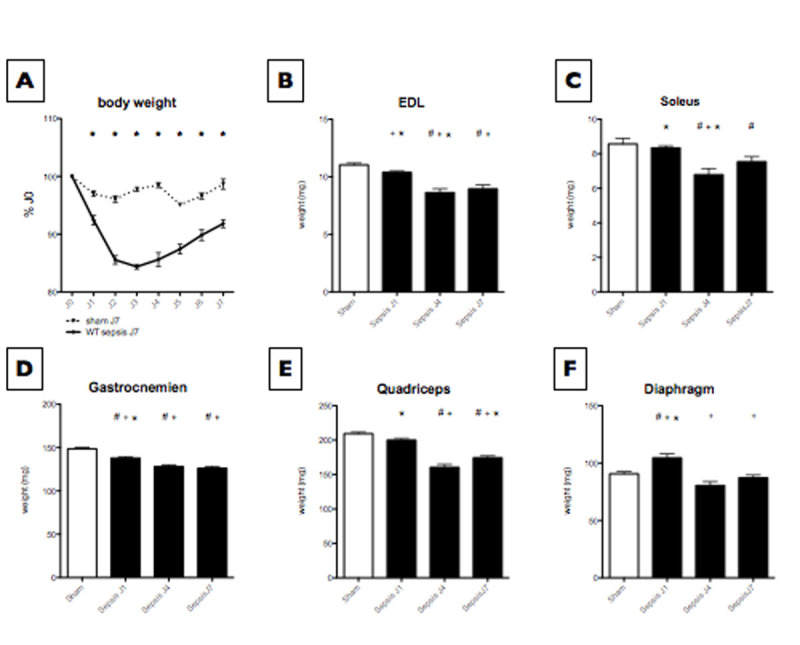
**Body and muscles weight.** A. Kinetic of animals' body weight (*p<0.05 versus JO). B, C, D, E Skeletal muscles weight (results are mean±SEM. * #' + p<0.05 versus sham, J1, J4 respectively). F Diaphragm weight (results are mean±SEM. * #' + p<0.05 versus sham, J1, J4 respectively).

**Figure 2:**
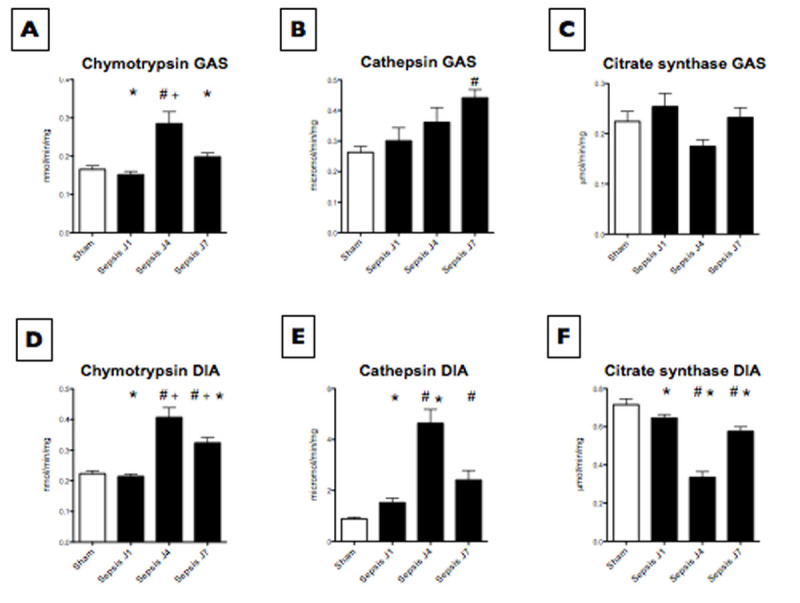
**Enzyme activity.** Enzyme activity in gastrocnemius (GAS) and diaphragm (DIA). Results are mean±SEM. * #' + p<0.05 versus sham, J1, J4 respectively

Loss of muscle mass and strength was observed as soon as day 1 following CLP and was maximum at day 4 for all skeletal muscles (Fig 1). In the gastrocnemius muscle, chymotrypsin-like activity of the proteasome was also higher at day 4 (Fig 2), whereas activity of cathepsin B+L, proteins involved in autophagy-lysosomal pathway, was maximum at day 7 (Fig 2). In the diaphragm muscle, chymoptrysin-like and cathespin B+L activities peaked at day 4. Citrate synthase activity, a marker of mitochondrial content, was minimal at day 4 both in gastrocnemius and diaphragm muscles.

## Conclusions

Our results confirm a major role for the proteasome in muscle wasting during sepsis. Interestingly, the increase in cathepsin B+L activity at day 7 in gastrocnemius muscle, when muscle mass had already started to recover, suggests that the autophagy pathway could participate to the recovery of skeletal muscle by inducing the clearance of damaged proteins and organelles. Such a late increase in cathepsin B+L activity was not observed in diaphragm muscle. The decrease in citrate synthase activity observed in both muscles may reflect mitochondrial impairment. These results should be completed by the ongoing analysis of other biological markers and histological data.

